# Profile of stingless bee honey and microbiota produced in West Sumatra, Indonesia, by several species (*Apidae, Meliponinae*)

**DOI:** 10.14202/vetworld.2024.785-795

**Published:** 2024-04-10

**Authors:** Sri Melia, Indri Juliyarsi, Yulianti Fitri Kurnia, Salam N. Aritonang, Rusdimansyah Rusdimansyah, Ade Sukma, Rizki Dwi Setiawan, Yudha Endra Pratama, Doni Supandil

**Affiliations:** 1 Department of Animal Products Technology, Faculty of Animal Science, Universitas Andalas, Padang, 25163, Indonesia; 2 Department of Animal Production, Faculty of Animal Science, Universitas Andalas, Padang, 25163, Indonesia; 3 Student of Doctoral Program, Faculty of Animal Science, Universitas Andalas, Padang, 25163, Indonesia; 4 Student of Magister Program, Faculty of Animal Science, Universitas Andalas, Padang, 25163, Indonesia

**Keywords:** antimicrobial, antioxidant activity, microbiota, physicochemical, stingless bee

## Abstract

**Background and Aim:**

Stingless bees are generally found in tropical countries, including Indonesia. In West Sumatra, stingless bees are known as *Galo-galo*, consist of several species with different characteristics; however, the properties of honey produced by stingless bees have not yet been explored. This study aimed to determine the physicochemical, antioxidant, and antimicrobial activities as well as the microbiota profile of stingless bee honey from the bee species *Heterotrigona itama*, *Geniotrigona thoracica*, *Tetrigona melanoleuca*, and *Tetrigona binghami* that are intensively developed in West Sumatra, Indonesia.

**Materials and Methods:**

Honey produced by the stingless bee species *H. itama*, *G*. *thoracica*, *T. melanoleuca*, and *T. binghami* originating in West Sumatra was examined in the present study. The physicochemical properties (Association of Official Analytical Chemists), antioxidant activity (2,2-diphenyl-1-picrylhydrazyl technique), total phenols (Folin–Ciocalteu method), antimicrobial activity (Agar-Well diffusion test), total lactic acid bacteria, and microbiota diversity were measured in stingless bee honey samples.

**Results:**

Stingless bee species significantly affected the physicochemical properties, antioxidant activity, total phenolic content, antimicrobial activity, and total lactic acid bacteria (p = 0.05), except for the crude fiber content. The carbohydrate profiles of honey produced by *H. itama* and *T. binghami* were dominated by monosaccharides, whereas those of honey from *T. melanoleuca* and *G. thoracica* were dominated by disaccharides. In terms of antioxidant activity (half maximal inhibitory concentration [IC_50_] value), there were no significant differences (p > 0.05) between honey from *H. itama*, *T. melanoleuca*, and *T. binghami*, but there were significant differences (p > 0.05) between honey from *G. thoracica*. The honey of *G. thoracica* and *T. melanoleuca* had the highest total phenolic content (65.65 ± 14.00 and 69.78 ± 8.06, respectively). In addition, honey from the four stingless bee species showed antimicrobial activity against the pathogenic bacteria *Escherichia coli*, *Salmonella*, *Staphylococcus aureus*, and *Listeria monocytogenes*. From the principal co-ordinate analysis (PCoA) results, it can be concluded that the microbiota profiles of the four stingless bee honey samples differed.

**Conclusion:**

The results showed that honey from *H. itama*, *G. thoracica*, *T. melanoleuca*, and *T. binghami* has different physicochemical characteristics, antioxidant activity, antimicrobial activity, and microbiota diversity. By knowing the content of this stingless bee honey, the results of this study can be used as information that this stingless bee honey has the potential as a functional food that is beneficial for health.

## Introduction

Stingless bees belonging to the Meliponinae subfamily are adept honey producers, yielding various valuable products akin to honey bees of the *Apis* genus [1]. The ectothermic nature of these honey bees makes them highly susceptible to environmental temperature fluctuations [2]. This agrees with the statement of Hilário *et al*. [3] that environmental factors, such as temperature, relative humidity, season, light intensity, and rainfall, greatly influence colony activity. Stingless bees are honey producers that are generally found in tropical countries, including Indonesia. In West Sumatra, stingless bees are known as *Galo-galo*. There are 18 stingless bee species in West Sumatra [4], with *Heterotrigona* *itama*, *Tetragonula laeviceps*, and *Geniotrigona thoracica* being the three most popular species for beekeeping, followed by *Tetragonula* *fuscobalteata*, *Tetragonula minangkabau*, and *Tetragonula* *testaceitarsis* [5]. Meliponiculture, the practice of maintaining stingless bee colonies, has gained traction, with certain species such as *H. itama* and *G. thoracica* emerging as preferred choices due to their superior honey yield and distinctive flavor profiles [6, 7].

Honey, a naturally occurring substance with a significant nutritional value, is produced by bees belonging to the *Apis* and *Meliponini* genera through the collection, alteration, and storage of nectar from floral sources, as well as sweet deposits derived from non-floral plants. The composition and quality of honey vary considerably depending on the botanical origin of the nectar and the current environmental and climatic conditions. The important role of honey in human health is related to its antimicrobial, anti-inflammatory, and antioxidant activities [8, 9]. Melia *et al*. [10] reported antimicrobial activity against several types of pathogenic bacteria in stingless bee honey originating from West Sumatra. Interestingly, honey is gradually gaining popularity as a source of alternative medicines [11] and continues to increase honey consumption. The characteristics of honey, such as viscosity, color, bitterness, sweetness, granularity, sourness, spiciness, fruitiness, and/or herbal aroma, which affect individuals’ choice for high-quality stingless bee honey [12], are starting to pay more attention to consumers. In addition to the physical factors mentioned above, the presence of bacteria affects the quality of honey. Casalone *et al*. [13] observed that several microbial communities present in propolis generated by *Apis mellifera* honey bees. The presence of microorganisms in honey greatly affects the quality and safety of honey for consumption; humidity and temperature strongly influence the growth rate of microorganisms in honey and eventually affect its storability. Eight bacterial phyla, 71 families, 155 genera, and 70 species of stingless bee honey from Selangor, Malaysia, have been identified [14]. In addition, honey is rich in fructose, and bacteria from the fructophilic lactic acid group can degrade fructose more easily [15]. Zawawi *et al*. [16] and Fletcher *et al*. [17] added that stingless bee honey also has a dominant type of disaccharide, namely trehalulose, in addition to monosaccharides (glucose and fructose).

This study was conducted to explore the physicochemical properties of honey sourced from four distinct stingless bee species co-existing in West Sumatra, namely *H. itama*, *G. thoracica*, *Tetrigona melanoleuca*, and *Tetrigona binghami*, while investigating their microbiota profiles and their potential as reservoirs of antioxidants and antimicrobial agents. Notably, certain species within the *Trigona* genus exhibit preferences for collecting either soft or hard resins. *H. itama* and *G. thoracica* demonstrate a preference for plants yielding soft resins, such as mango, jackfruit, and dammar trees, whereas *T. binghami* and *T. melanoleuca* favor hard resin sources, particularly pine tree honey.

## Materials and Methods

### Ethical approval

This study does not require ethical approval, as the observation does not involve experimental design that includes any living beings. Additionally, the study’s focus was on the final product of a stingless bee (honey), without causing harm to the bees in the process.

### Study period and location

Honey samples were collected from January to June 2023 at a stingless beekeeping farm at the Faculty of Animal Science, Universitas Andalas. The average temperature was 26°C (78.8°F), with a relative humidity of 94% in the hottest month (January–June).

### Materials

The honey (n = 12) examined in this study was produced by stingless bee species *H. itama*, *G. thoracica*, *T. melanoleuca*, and *T. binghami* originating in West Sumatra and being reared in the same location, namely, a stingless beekeeping farm at the Faculty of Animal Science, Universitas Andalas. These species were identified by Rusdimansyah, who conducted the identification based on the guidance book ‘Indo-Malayan Singless Bee: Pictorial Identification Guide and Composite Algorithm’. Before honey samples were analyzed, they were stored in sterile falcon tubes at 4°C in a refrigerator.

### Physicochemical analyses

#### Moisture content, water activity, and pH

The moisture content of honey samples was measured according to the Association of Official Analytical Chemists (AOAC) [18] using the oven method. A water activity meter (Novasina Labmaster Aw, Switzerland) was used to measure the water activity (aw). Each 10 g sample was diluted in 75 mL of distilled water, homogenized, and read at room temperature after equilibrium [19].

The pH value of each honey sample was also measured according to AOAC [18] using a pH meter (Hanna, Romania). The device was calibrated using a buffer solution with pH values of 3 and 7. Five milliliters of the sample were added to 10 mL of distilled water and then read after equilibrium was achieved.

#### Viscosity

The viscosity was determined according to Oroian [20] with a slight modification using a viscometer (Brookfield LV, USA) equipped with spindles number 3 at 25°C and 50 rpm. For each sample, the test was carried out 5 times.

#### Total sugar content

Total sugar content was quantified using the phenol-sulfuric acid method, with glucose serving as the standard at various concentrations (0, 1, 2.5, 5, 10, 25, 50, and 100 ppm). Honey samples were dissolved in distilled water at a ratio of 0.5 g/20 mL. This process was repeated until the total volume reached 100 mL. The solution was then filtered using Whatman filter paper. Subsequently, 1 mL of the aforementioned solution was combined with 1 mL of the 5% phenol solution and 5 mL of the 96% sulfuric acid solution. The resulting solution was then subjected to vortexing and left undisturbed until it equilibrated to ambient temperature. Subsequently, it was quantified at a specific wavelength of 490 nm using an ultraviolet-visible (UV-Vis) spectrophotometer (Shimadzu, Japan) [21].

#### Crude fiber content

The fiber content was measured using an enzymatic gravimetric method. For this purpose, 1 g of sample was added to 50 mL of phosphate buffer (pH 6.0) and 50 μL of termamyl. The solution was heated in a water bath maintained at 95°C–100°C for 15 min. The solution was stirred at intervals of 5 min. The solution was then allowed to cool. Samples at pH 7.5 were then added to 100 μL of protease enzyme and incubated for 30 min at 60°C. The mixture was then adjusted to pH 4.5, added to 200 μL of amyloglucosidase enzyme, and incubated for 30 min at 60°C. The solution was then settled for 60 min by adding 280 mL of 95% ethanol. The resulting precipitates were weighed and filtered using Whatman filter paper, washed twice with 78% ethanol and once with acetone, and then placed in an oven at 105°C for 24 h. The results were weighed as the weight of dry residue. Furthermore, the ash content and residual protein content of the residue were analyzed. The crude fiber content was calculated by subtracting the dry residue from the ash and protein residues, divided by the sample weight, and represented as a percentage [18].

#### Carbohydrate profile

The carbohydrate profile was determined according to Zhang et al. [22] with some modifications; the adjustment in the eluent uses only acetonitrile in water, with column temperature kept at 35°C and flow rate constant at 1 ml/min. Samples weighing 0.5 g were dissolved in demineralized water in a 10 mL volumetric flask. Each 1 mL sample solution was then diluted in a 10 mL volumetric flask. Samples were measured using high-performance liquid chromatography with an external standard method (500–3000 ppm).

**Table:** 

	Condition
Column	Restek’s Ultra Amino 150×4.6 mm
Eluent	Acetonitrile-Water (75-25)
Flow rate	1 mL/min
Detector	RID (35°C)
Standard	Fructose (monosaccharide) and maltose (disaccharide)

#### Total phenolic content

Total phenols in honey samples were quantified using the Folin-Ciocalteu method. 1 g of each honey sample was combined with 10 mL of distilled water and subsequently filtered. Various amounts of honey solution were combined with the Folin-Ciocalteu reagent at 0.2 N. After 5 min, 1.5 mL of sodium carbonate (Na_2_CO_3_) was added to the solution. Subsequently, the combination was incubated at ambient temperature for 30 min. In addition, the absorbance of the reaction mixture was measured at 670 nm. A calibration curve was calibrated using gallic acid as a standard [23].

#### Antioxidant activity

Free radical scavenging activity was determined using the 2,2-diphenyl-1-picrylhydrazyl (DPPH) technique as described by Mulugeta and Belay [24], with minor adjustments. Honey samples were diluted with distilled water. Various quantities of the honey solution were then combined with 2 mL of a 0.1 mM DPPH solution in methanol. Subsequently, the concoction was incubated in the dark for 30 min. Subsequently, the absorbance was quantified at 520 nm using a UV-Vis spectrophotometer (Shimadzu). The analysis was conducted in three replicates, and the free radical scavenging activity percentage for each sample was determined using the following equation:

% inhibition = (Abs (control) - Abs (sample)/Abs (control)) × 100.

where Abs (control) and Abs (sample) are the absorbances of the control and the samples, respectively. Antioxidant activity is expressed by the half-maximal inhibitory concentration (IC_50_) value, which is the concentration of the sample required to achieve 50% inhibition.

#### Antimicrobial activity

The antibacterial activity of honey samples at various concentrations against four strains of bacteria, including two Gram-positive strains (*Staphylococcus aureus* and *Listeria monocytogenes*) and two Gram-negative strains (*Salmonella* and *Escherichia coli*), was studied. Diluted bacterial culture (1 mL) was evenly distributed on individual Petri dishes prepared with Mueller–Hinton agar medium. A total of five wells measuring 0.6 cm in diameter were created in the inoculated agar. These wells were carefully cut using a sterilized cork borer. The wells were then filled with the test samples using a sterile dropper. Subsequently, the plate was incubated at 37°C for 24 h. The zone of inhibition was measured at the end of the incubation period [25].

#### Total lactic acid bacteria

1 mL of honey sample was introduced into 9 mL of sterile water and gradually diluted. As a result, the solution was diluted, which resulted in concentrations ranging from 10^–1^ to 10^–8^ times lower than those of the original stock solution. Subsequently, 1 mL of the diluted solution was transferred into a Petri dish, and 15 mL of de Man, Rogosa, and Sharp agar media were added. The specimens were subjected to homogenization and then solidification. Subsequently, every Petri dish was incubated at 37°C for 24–48 h [26].

#### Color

To measure the color properties of the honey samples, the color of the samples was determined using a colorimeter (ColorFlex, Hunterlab, VA, USA). The L*, a*, and b* values are the degrees of lightness to darkness, redness to greenness, and yellowness to blueness, respectively [27].

#### 16s ribosomal RNA (rRNA) gene

The 16s rRNA amplification gene was taken from two of nine hypervariable regions using the amplicon sequencing technique [28]. The primer targeting the V3-V4 hypervariable region was polymerase chain reaction (PCR)-amplified through primer sets 341F-806R (341F 5’-CCTAYGGGRBGCASCAG-3’ and 806R 5’-GGACTACNNGGGTATCTAAT-3’) [29]. The PCR amplification was performed as follows: 95°C for 3 min, 25 cycles (95°C for 30 s, 55°C for 30 s, 72°C for 30 s), 72°C for 30 s, and stored at 4°C. The PCR fragment size was approximately 470 bp, as determined by the Agilent 2100 Bioanalyzer (Agilent Technology, Santa Clara, USA). The labeling of amplicons of disparate dual barcodes is supported by the Nextera XT index kit (Illumina, San Diego, USA).

### Statistical analysis

Sukma *et al*. [30] compiled the data archived interpretation PCR amplifications. The archived raw sequence sample data were interpreted using Mothur V1.2.7,http://ccb.jhu.edu/software/FLASH/for splitting barcodes [31]. The demultiplexed sequence was clustered into Operation Taxonomic Units (OTUs) based on the 97% similarity sequence from the Ribosomal Database Project classifier (Uparse v7.0.1001, http://drive5.com/uparse/) [32]. OTU interpretation was assigned by the GreenGene Database (http://greengenes.lbl.gov/cgi-bin/nph-index.cgi)33]. The principal component analysis (PCA) was supported by the Statistical Package for the Social Sciences v23.0 (IBM Corp., NY, USA) and PCoA analysis was displayed by WGCNA package, stat packages, and ggplot2 package in R software(Version 2.15.3). [34].

## Results and Discussion

### Physicochemical properties of stingless honey

#### Moisture content and water activity (a_w_)

Table-1 shows the moisture contents of honey from the four stingless bee species. The results of the statistical analysis indicated no significant difference (p > 0.05) in the moisture content of stingless bee honey among *H. itama*, *T. melanoleuca*, and *T. binghami*, except for honey from *G. thoracica*. The moisture content of honey from *H. itama* and *G. thoracica* obtained in this study had moisture contents of 19.49–26.28 g/g and 21.32–33.93 g/g, respectively, in honey produced by the same species of bees originating from Malaysia, as reported by Shamsudin *et al*. [35]. Meanwhile, honey from *T. melanoleuca* had much lower moisture content than that reported by Chuttong *et al*. [36], which reached 43 g/g.

**Table-1:** Physicochemical properties of stingless bee honey.

Stingless bee species	Moisture content (g/g)	Water activity	Viscosity (cps)	pH	Crude fiber (g/g)	Total sugar (%)
*H. itama*	23.46 ± 0.03^a^	0.65 ± 0.56^a^	1072 ± 0.93^a^	3.94 ± 0.18^a^	0.48 ± 0.10	66.24 ± 0.44^a^
*G. thoracica*	30.02 ± 0.15^b^	0.72 ± 0.13^b^	431 ± 0.45^b^	3.07 ± 0.18^b^	0.42 ± 0.30	67.97 ± 0.90^a^
*T. melanoleuca*	24.26 ± 0.34^a^	0.68 ± 0.57^c^	968 ± 0.22^c^	3.04 ± 0.21^b^	0.47 ± 0.51	98.02 ± 0.65^b^
*T. binghami*	23.86 ± 0.50^a^	0.66 ± 0.27^c^	869 ± 0.36^c^	3.12 ± 0.24^b^	0.41 ± 0.02	88.02 ± 0.50^b^

*The difference in superscripts in the same column indicates a significant difference (p<0.05), *H. itama*=*Heterotrigona itama*, *G. thoracica*=*Geniotrigona thoracica*, *T. melanoleuca*=*Tetrigona melanoleuca*, *T. binghami*=*Tetrigona binghami*

The moisture content of honey is strongly influenced by the intrinsic characteristics of each bee species [19], which are related to floral resources, climatic conditions, and post-extraction handling [37, 38]. Based on the guidelines outlined in the International Food Standards for Honey Alimentarius [39], the moisture level of honey should not exceed 20 g/g, which means that all honey from bee species examined in this study did not meet international regulatory requirements. However, the Indonesian government specifically enforced standards to guarantee the quality of stingless bee honey, with a maximum moisture content of 27.5 g/g [40]. Therefore, the honey produced by the *G. thoracica* bee alone does not meet these requirements.

Regarding water activity, honey produced by *G. thoracica* exhibited the highest water activity, with a statistically significant difference (p < 0.05) from honey produced by the remaining three species (Table-1). Because honey from *G. thoracica* has the highest moisture content, water activity is closely related to the moisture content of the sample. Although water activity parameters are not regulated in honey standards, understanding honey’s water activity can assist in investigating its quality, especially in estimating its microbiological aspects [41]. High moisture content and water activity can cause rapid microbial growth, especially that of osmotolerant yeast, which can still grow at a water activity of at least 0.6 [42].

#### pH

Four stingless bee species produced honey with a pH ranging from 3.04 to 3.94, indicating acidic characteristics (Table-1). The results showed a significant difference (p < 0.05) between honey from *H. itama* and the other three types of samples in terms of pH. Overall, all honey samples in this study had a lower mean pH value than honey from 11 stingless bee species from Thailand (3.6 ± 0.20), 4 stingless bee species from Ethiopia (3.7 ± 0.15), and 11 stingless bee species from Brazil (3.89 ± 0.66) [19, 36, 38], indicating that stingless bee honey originating from West Sumatra, Indonesia, has more acidic characteristics. Similarly, Shamsudin *et al*. [35] reported that the pH values of honey from *H. itama* and *G. thoracica* originating in Malaysia ranged between 3.19 and 3.55 and 3.17 and 3.40, respectively.

This wide range of pH is influenced by differences in floral resources and bee species [38], harvest time, and organic acid content [43]. All honey samples in this study had a pH value below 7.0, meaning that they were acidic, requiring good storage stability and shelf life [44].

#### Viscosity

In this study, honey from all four stingless bee species produced extremely varied viscosities, and different stingless bee species resulted in significant differences (p < 0.05) in the viscosity of honey produced (Table-1). The viscosity value of each honey sample was consistent with the moisture content of the honey. Honey from *H. itama* had the lowest moisture content and the highest viscosity. On the other hand, honey produced by *G. thoracica*, which has the highest moisture content, has the lowest viscosity. Honey from *T. melanoleuca and T. binghami* did not differ significantly (p > 0.05) in viscosity.

The results of this study are similar to those of honey from *G. thoracica* from Malaysia, which has a viscosity of 257 cp [45]. However, the viscosity of the honey produced by *H. itama* in this study was much higher than that reported by Baroyi *et al*. [46], in which the viscosity was only 330 cps with a moisture content of 25.82 g/g. Differences in the viscosity of the honey produced are influenced by the diversity of the floral resources. When bees obtain nectar with a high sugar content, the viscosity of the honey they produce will be affected [47]. In addition, viscosity can change during storage and heating processes [45, 48]. The viscosity of honey is also influenced by the content and composition of sugar, as well as the number of types of colloids that affect its texture and are related to its processing and consumption [42].

#### Total sugar

A significant difference (p = 0.05) in the total sugar content of honey was observed in the stingless bee species (Table-1). *T. melanoleuca* honey had the highest total sugar content, followed by *T. binghami*, *G. thoracica*, and *H. itama*. Overall, the total sugar content of honey in this study was higher than that of honey from *G. thoracica* (44.98%–61.37%), *H. itama* (47.25%–55.61%), and *T. melanoleuca* (15.0%) originating from Malaysia [35] and Thailand [36]. The sugar content and composition of honey are highly influenced by the source of nectar, geographical origin, and climate [49].

#### Crude fiber content

The crude fiber content in stingless bee honey in this study ranged from 0.41 to 0.48 g/g (Table-1), indicating that different species of stingless bees did not significantly influence (p > 0.05) the fiber content of the honey produced. These values are lower than honey from various *Apis* species, ranging from 1.99 to 2.76 g/g [50]. Furthermore, Abdullah *et al*. [51] reported that the crude fiber content of propolis from *H. itama*, another product of this stingless bee species, is 0.30 g/g. These results indicate that honey and propolis from stingless bees have similar crude fiber contents. The low fiber, protein, and carbohydrate contents of propolis can prevent fermentation, resulting in a short shelf life.

#### Color

The visual appearance of a product is a significant determinant of customer preference, taking into account aroma and texture. According to Safi *et al*. [52], color is an important factor that consumers must pay attention when choosing honey. According to Solayman *et al*. [53], the color of honey ranges from yellow to dark color. It depends on the mineral content of honey, pollen, and several pigments such as carotenoids, chlorophyll flavonoids, and polyphenols [54]. As shown in Table-2, there was a significant difference (p < 0.05) in the color measurement of stingless bee honey among the four species. These results indicate that the honey produced in this study has a relatively dark color. *G. thoracica* honey had the lowest L* value, indicating a darker color. High antioxidant activity has been reported in dark-colored honey [55].

**Table-2:** L*, a*, b* value of stingless bee honey.

Stingless bee species	L*	a*	b*
*H. itama*	13.78 ± 0.05^c^	3.76 ± 0.03^d^	10.69 ± 0.02^c^
*G. thoracica*	7.49 ± 0.08^a^	-2.55 ± 0.02^a^	3.10 ± 0.11^a^
*T. melanoleuca*	18.19 ± 0.12^d^	1.40 ± 0.10^c^	9.86 ± 0.14^b^
*T. binghami*	10.64 ± 0.02^b^	-1.44 ± 0.05^b^	3.10 ± 0.01^a^

* The difference in superscripts in the same column indicates a significant difference (p<0.05), *H. itama*=*Heterotrigona itama*, *G. thoracica*=*Geniotrigona thoracica*, *T. melanoleuca*=*Tetrigona melanoleuca*, *T. binghami*=*Tetrigona binghami*

Color is one of the major characteristics of honey that can be used to identify floral resources [56]. The color of the honey ranges from light yellow to dark yellow, with occasional green or red shades. In extreme cases, honey may be black in color [57]. The color of honey depends on its moisture content, saccharides, minerals, pollen, and polyphenolic compounds [51]. Honey originating from *H. itama* and *G. thoracica* from Malaysia has a much higher L* value (73.98–92.57) compared with that obtained in the present study, indicating that honey has a much brighter color [35]. It can be attributed to the different floral resources, bee species, and honey production locations. Differences in honey color can also be attributed to storage duration and temperature [42].

#### Carbohydrate profile

Carbohydrates are one of the main components of honey. The carbohydrate profiles of the stingless honey samples examined in this study are presented in Table-3. Fructose and maltose were the standard sugars used for monosaccharides and disaccharides. Honey produced by *H. itama* and *T. binghami* had high monosaccharide sugar contents. Honey from *G. thoracica* and *T. melanoleuca* had a higher disaccharide sugar content than honey from the other two species. A previous study by Nordin *et al*. [58] revealed that the maltose content in stingless bee honey ranges from 0.6 g/100 g to 53 g/100 g. Furthermore, Solayman *et al*. [53] explained that in addition to reducing sugars, maltose and sucrose are also present in stingless bee honey, although the amounts are very low compared with those of fructose and glucose.

**Table-3:** Carbohydrate profile of stingless bee honey.

Stingless bee species	Monosaccharides (%)	Disaccharides (%)
*H. itama*	47.4	11.0
*G. thoracica*	10.0	44.7
*T. melanoleuca*	14.9	44.7
*T. binghami*	37.7	18.3

*H. itama*=*Heterotrigona itama*, *G. thoracica*=*Geniotrigona thoracica*, *T. melanoleuca*=*Tetrigona melanoleuca*, *T. binghami*=*Tetrigona binghami*

#### Antioxidant activity and total phenolic content

In this study, honey from *G. thoracica* had the highest IC_50_ value and significantly differed (p > 0.05) from the other three honeys, whereas there was no significant difference (p > 0.05) in antioxidant activity among honeys from *H. itama*, *T. melanoleuca*, and *T. binghami* (Table-4). These results indicate that the honey from *G. thoracica* had the lowest antioxidant activity among the samples. According to Bastos *et al*. [59], stingless bee honey exhibits higher antioxidant activity than honey produced by *A. mellifera* bees because of its higher content of phenolic compounds and flavonoids. Neupane *et al*. [60] reported a strong correlation between antioxidant activity and total phenol content in honey. Shamsudin *et al*. [35] found that gelam honey and starfruit honey produced by *H. itama* exhibited the lowest and highest IC_50_ values, respectively. Gelam honey demonstrated a much lower IC_50_ value of 32.58 mg/mL than starfruit honey (105.53 mg/mL), indicating a higher level of antioxidant activity. Silva *et al*. [61] reported a relatively low IC_50_ value for honey derived from *Melipona subnitida* bees in Brazil, which ranged from 10.60 to 12.90 mg/mL. Honey with a lower IC_50_ value demonstrated superior antiradical activity compared with those with a larger IC_50_ value [62].

**Table-4:** Antioxidant activity (IC_50_) and total phenol contents of stingless bee honey.

Stingless bee species	IC_50_ value (mg/mL)	Total phenol (mg GAE/g)
*H. itama*	21.6 ± 0.36^a^	52.61 ± 0.24^b^
*G. thoracica*	31.2 ± 0.62^b^	65.65 ± 14.00^c^
*T. melanoleuca*	22.6 ± 0.47^a^	69.78 ± 8.06^c^
*T. binghami*	23.4 ± 1.02^a^	30.43 ± 5.90^a^

*The difference in superscripts in the same column indicates a significant difference (p<0.05), *H. itama*=*Heterotrigona itama*, *G. thoracica*=*Geniotrigona thoracica*, *T. melanoleuca*=*Tetrigona melanoleuca*, *T. binghami*=*Tetrigona binghami*, GAE=Gallic acid equivalent

In terms of total phenolic content, honey from *G. thoracica* and *T. melanoleuca* had the highest total phenolic content and significantly differed from honey from *H. itama* and *T. binghami* (p < 0.05; Table-4). Selvaraju *et al*. [63] found that the total phenolic content of *H. itama* and *G. thoracica* honey ranged from 65.67 to 114.38 mg gallic acid equivalent (GAE)/kg. The total phenolic content of six stingless bee honey samples from Malaysia (*H. itama*) ranged from 27.33 to 55.86 mg GAE/100 g [28]. Moreover, honey contains gallic, coniferic, benzoic, and trans-cinnamic phenolic compounds [64].

The phenolic content of honey is influenced by various other factors, such as harvest season, climatic conditions, and processing parameters [65]. Shubharani *et al*. [25] revealed differences in the total phenolic content of honey from different floral resources, with acacia honey produced by *G. thoracica* having 55.86 mg GAE/100 g and *gelam* honey produced by *H. itama* having 52.25 mg GAE/100 g total phenolic contents. According to Gheldof *et al*. [66], honey contains phenolic acids (benzoic acid and cinnamic acid) and flavonoids (flavanone and flavanol) that significantly contribute to the therapeutic capacity of honey, which varies greatly depending on the source of the flowers.

#### Correlation between honey from stingless bees and the physicochemical properties

**Figure-1:**
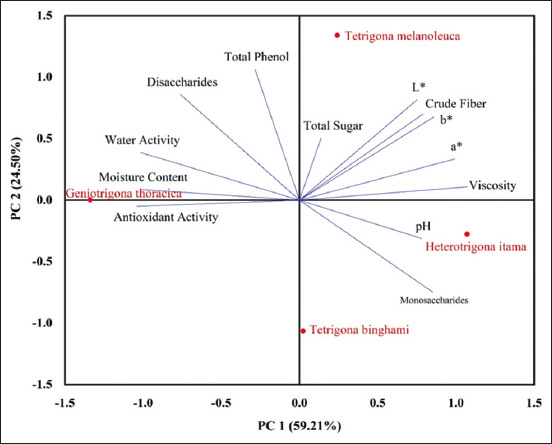
Principal component analysis of physicochemical analysis.

PCA showed a total variation of 83.71%, with each component contributing 59.21% and 24.50% to first principal component (PC1) and second principal component (PC2), respectively (Figure-1). Moisture content and water activity exhibited a strong negative correlation, whereas viscosity exhibited a strong positive correlation with PC1. In contrast, the total phenolic content showed a strong positive correlation with PC2. In this study, moisture content and water activity were strongly negatively correlated with viscosity (r = 0.97 and 0.92, respectively) but strongly positively correlated with antioxidant activity (IC_50_) (r = 0.99 and 0.93, respectively).

Overall, PCA results revealed that stingless bee honey from *T. binghami* and *H. itama* lies within the same quadrant, indicating similar characteristics with high pH and monosaccharide content. Stingless honey from *G. thoracica* occupies a distinct quadrant characterized by high moisture content and water activity, whereas honey from *T. melanoleuca* occupies another quadrant characterized by high brightness levels.

#### Antimicrobial activity

All four types of stingless bee honey exhibited significant antibacterial activities (p < 0.05) against various pathogenic bacteria, including *E. coli*, *Salmonella*, *S. aureus*, and *L. monocytogene*s. *G. thoracica* honey showed the highest inhibitory activity against *Salmonella* and *S. aureus* compared with the other three honey samples. In honey produced by *T. binghami*, the greatest inhibitory activity against *L. monocytogenes* was observed (Table-5).

**Table-5:** Antimicrobial activity of stingless bee honey.

Stingless bee species	*E. coli* (mm)	*Salmonella* (mm)	*S. aureus* (mm)	*L. monocytogenes* (mm)
*H. itama*	13.0 ± 0.62^a^	12.5 ± 0.09^b^	12.0 ± 0.15^a^	11.5 ± 0.99^b^
*G. thoracica*	15.0 ± 0.33^a^	16.5 ± 0.56^c^	17.0 ± 0.31^b^	11.5 ± 0.34^b^
*T. melanoleuca*	11.0` ± 0.83^a^	10.5 ± 0.48^a^	10.0 ± 0.61^a^	10.0 ± 0.22^a^
*T. binghami*	12.0 ± 0.34^a^	13.0 ± 0.23^b^	11.5 ± 0.12^a^	12.0 ± 0.12^c^

*The difference in superscripts in the same column indicates a significant difference (p<0.05), *H. itama*=*Heterotrigona itama*, *G. thoracica*=*Geniotrigona thoracica*, *T. melanoleuca*=*Tetrigona melanoleuca*, *T. binghami*=*Tetrigona binghami*, *E. coli*=*Escherichia coli*, *S. aureus*=*Staphylococcus aureus*, *L. monocytogenes*=*Listeria* *monocytogenes*

Similar results were reported by Ng *et al*. [67], who reported that honey from *H. itama* and *G. thoracica* had antibacterial activity against *E. coli* and *S. aureus*. Stingless bees also showed greater spectrum inhibitory activity against Gram-positive (*S. aureus*) and Gram-negative bacteria (*E. coli* and *Klebsiella*) compared to *Apis* honey bees [68]. Moreover, Shubharani *et al*. [25] discovered that honey originating from Karnataka prevented the proliferation of *E. coli* but did not inhibit the growth of *S. aureus*.

The level of antibacterial activity in honey varies owing to factors such as bee species, nectar supply, and intrinsic properties such as osmotic effects, phytochemical acidity, and hydrogen peroxide [11]. However, the main factor contributing to antimicrobial activity is the presence of hydrogen peroxide in honey, which is generated through glucose oxidation catalyzed by glucose oxidase [69, 70].

The stingless bee honey produced by *Homotrigona fimbriata* exhibited the most potent antimicrobial action by inhibiting the growth of five bacterial strains, including *S. aureus*, *Serratia* *marcescens*, *Bacillus* *subtilis*, *E. coli*, and *Alcaligenes faecalis*. *H. itama* honey was active against *S. aureus* and *B. subtilis*, whereas *G. thoracica* and *T. binghami* honey only had antibacterial activity against *S. aureus* [14].

#### Total amount of lactic acid bacteria

There was a significant difference (p < 0.05) in the total lactic acid bacteria contained in stingless bee honey in this study, as shown in Table-6, where honey from *G. thoracica* had the lowest total lactic acid bacteria and significantly differed from the other samples. Stingless bee honey contains *Lactobacillus plantarum* SNT13T as a probiotic candidate [71]. *Lactobacillus malefermentans* is the dominant bacterium found in stingless bee honey from TATI Agro Farm in Semenyih, Selangor, Malaysia [14].

**Table-6:** Total lactic acid bacteria of stingless bee honey.

Stingless bee species	Total lactic acid bacteria (10^8^ CFU/mL)
*H. itama*	8.2 ± 0.82^b^
*G. thoracica*	5.9 ± 1.05^a^
*T. melanoleuca*	7.7 ± 1.22^b^
*T. binghami*	8.1 ± 0.78^b^

*The difference in superscripts in the same column indicates a significant difference (p<0.05), *H. itama*=*Heterotrigona itama*, *G. thoracica*=*Geniotrigona thoracica*, *T. melanoleuca*=*Tetrigona melanoleuca*, *T. binghami*=*Tetrigona binghami*, CFU=Colony forming unit

#### Microbiota in honey

**Figure-2:**
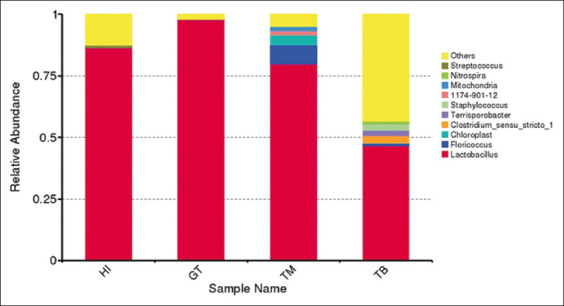
Variations in the genus of bacteria in stingless bee honey.

A total of 802.943 reads obtained from the four stingless bee honey samples were clustered into 1169 OTUs using the Ribosomal Database Project classifier at 97% identity [32]. The SILVA rRNA database was used to align each OTU [33]. OTUs were grouped into 37 phyla, 432 genera, and 171 species. As shown in Figure-2, *Lactobacillus* dominated the diversity of bacteria in stingless honey bee samples, especially *G. thoracica*-produced honey.

**Figure-3:**
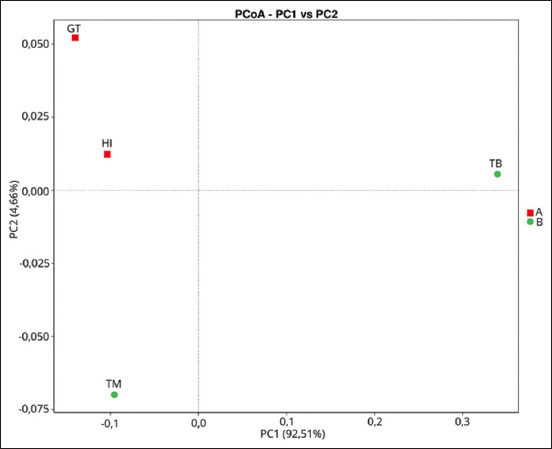
The PCoA of the weighted UniFrac (beta diversity) in the lactic acid bacteria of stingless bee honey.

The highest OTU was observed in *H. itama*, where up to 1005 species were identified. Alpha diversity of bacterial profile of stingless bee honey was described using Shannon (evenness) and Simpson (richness) indices. Richness refers to the total number of bacterial species in a community, whereas evenness refers to the abundance of bacterial species in the community [72, 73]. As shown in Table-7, *T. binghami* produced the highest values in terms of richness and evenness. Figure-3 displays the PCoA of the weighted UniFrac (beta diversity) in the lactic acid bacteria of stingless bee honey. The absence of diversity in the same cluster indicates that all honey samples have similar bacterial communities. All honey samples are classified on the basis of their resin content.

**Table-7:** Richness and evenness of stingless bee honeys.

Sample name	Observed species	Shannon (Evenness)	Simpson (Richness)	Chao1 index	ACE index	Good’s coverage	PD whole tree
*H. itama*	1005	1.834	0.278	1117.734	1134.378	0.998	94.738
*G. thoracica*	68	0.877	0.215	89.111	89.425	1	14.132
*T. melanoleuca*	78	2.559	0.607	78.75	79.996	1	16.22
*T. binghami*	275	3.013	0.748	298.786	296.928	1	33.128

*H. itama*=*Heterotrigona itama*, *G. thoracica*=*Geniotrigona thoracica*, *T. melanoleuca*=*Tetrigona melanoleuca*, *T. binghami*=*Tetrigona binghami, ACE=Abundance-based Coverage Estimator, PD=*Phylogenetic Diversity

## Conclusion

Honey produced by four species of stingless bees from West Sumatra, Indonesia, differed in various physicochemical properties, antioxidant and antimicrobial activities, and total phenolic and lactic acid bacteria content, except for crude fiber. Honey produced by *G. thoracica* exhibited the highest moisture content, water activity, and pH, whereas honey from *H. itama* exhibited the highest viscosity. Honey from different species showed similar antioxidant activities but different phenolic content and carbohydrate profiles. PCA of the physicochemical properties of stingless bee honey revealed that *T. binghami* and *H. itama* honey share similar characteristics, whereas *G. thoracica* and *T. melanoleuca* honey exhibit distinct individual characteristics. All honey also showed good antimicrobial activity against the pathogenic bacteria *E. coli*, *Salmonella*, *S. aureus*, and *L. monocytogenes*. PCoA showed differences in the microbiota profile of each type of stingless bee honey examined in this study. However, the characteristics of the honey samples during storage remain unknown. Therefore, further studies are needed to determine changes in the characteristics and quality of stingless bee honey during storage.

## Data Availability

All the data, including the information presented in the text, tables, and figures, are provided in the manuscript.

## Author’s Contributions

SM, AS, and RR: Concept and design. AS, SM, YFK, and SNA: Analysis and interpretation. SM, YEP, and DS: Data collection. SM, RR, AS, and RDS: Data acquisition and analysis and drafted the manuscript. SNA and IJ: Critical revision of the article. YEP: Statistical analysis. SM: Supervised the study. All authors have read, reviewed, and approved the final manuscript.
